# Supplementary material for: Metabolically healthy obesity: Inflammatory biomarkers and adipokines in elderly population
Source: PLoS One. 2022 Jun 9;17(6):e0265362. doi: 10.1371/journal.pone.0265362 (PMC9182320; doi:10.1371/journal.pone.0265362)
Supplement: S1 Protocol — (DOCX) [file pone.0265362.s001.docx]

**Title:** **Obese metabolically healthy elderly population: -omic studies (epigenetics, metabolomics, metagenomics) and its relationship with environmental pollutants**

**Protocol/serial number:** PI18/00766

**Chief researcher:** Ricardo Gómez-Huelgas

**Abstract**:

Metabolically Healthy Obese individuals (MHO) elderly are a minority phenotype and special, seemingly protected from the cardiometabolic disorders associated to excess adiposity. The aim of this study is to test whether the change of lifestyle by promoting physical exercise and recommendations of healthy Mediterranean-style diet (without caloric restriction) in elderly MHO is associated with changes in insulin sensitivity and whether modifies the metabolomic mechanisms, epigenetic patterns and gut microbiota in this population. For this purpose, 120 subjects will be recruited, with aged 65-80 years, with: a) a body mass index (BMI) ≥30-<35 kg/m2 mantained for at least 10 years before the recruitment, b) a HOMA-IR index <2.73, and c) ≤1 of the following 4 criteria: blood pressure ≥135/85 mmHg, fasting blood glucose ≥100 mg/dL, HDL-cholesterol <50 mg/dL in women and <40 mg/dL in men, triglycerides ≥150 mg/dL. The lipid profile, inflammatory biomarkers (hsPCR, IL6, TNFa, fibrinogen) and adipokines (adiponectin, leptin) will be analyzed. Epigenetic (methylation), metabolomic, and metagenomic (gut microbiota) studies, and of will be carried out and their possible relationship with different environmental pollutants will be analyzed.

**Background and current avalaible evidence**:

Obesity is a complex and heterogeneous syndrome that can be defined as a chronic disease mainly caused by the increament of fat tisue. The main cause of obesity is an imbalance in calories consumed and calories expended, although metabolic, physiology, genetic, celular, molecular, cultural and social factors may be involved (**1**). Globally, there is a trend toward consuming high-energy foods that are usually high in free sugars and fat, as well as a trend toward performing less physical activity. These trends are mainly caused by changes in social and environmental factors. A subset of these subjects is known as “metabollically healthy obese” (MHO) and, despite excess fat tissue, they are defined by a favorable metabolic profile. This profile is characterised by a high insulin sensitity, low visceral fat, low liver steatosis, normal blood pressure and favorable lipid, inflammatory, hormonal and inmune profiles. Although the mechanisms that could explain this benign metabolic profile of MHO subjects are still not well understood, the evidence suggests that certain factors such as the pattern of visceral fat deposition, birth weight, adipocyte size or expression of certain genes that regulate the differentiation and expansion of adipose tissue could be important (**2**).

There is currently great interest in investigating the MHO phenotype, as it can provide key data for a better understanding of the pathophysiological mechanisms of obesity. However, preventive interventions in this group remain controversial. On the one hand, it has been described that MHO individuals represent a subgroup of patients with better clinical evolution, since they will have a lower risk of developing cardiovascular disease or type 2 diabetes than the rest of obese subjects (**3-5**). They may not even get metabolic benefits from weight loss (**6,7**) or physical activity programs (**8**). Given its favorable cardiometabolic prognosis (**3**), a less intensive therapeutic approach could be justified (**9,10**). However, there are discrepancies regarding the clinical benignity of the MHO phenotype since it has been suggested that it can only constitute a simple transitional form to an obese phenotype with cardiometabolic alterations, although it is true that the phenotype MHO can be found in people of all ages (**11,12**).

Our research group has reported a high prevalence of obesity and metabolic syndrome among the adults in our population (Málaga, Andalusia) (**13,14**). However, the prevalence of MHO that we have documented is even lower than the reported by other authors. According to our data, only 9.6% of the obese population (2.2% of the adult population) would have a MHO phenotype (**15**). The MHO phenotype has been reported to be more prevalent in women and decreases with age (**3,4,16**). In our population, the MHO subjects had an average age almost ten years younger than obese patients with metabolic alterations (**15**), data confirmed by other authors (**17**). This might suggest that part of the MHO population could end up developing a cardiometabolic risk phenotype over time.

On the other hand, several epidemiological studies have observed that patients with overweight or moderate obesity have a lower risk of death and cardiovascular events than subjects with normal or low BMI (**18**), although this fact can be attributed to a form of reverse epidemiology. This phenomenon, called the "obesity paradox", is common in the elderly, in whom overweight or grade I obesity are associated with longer survival (**19**). BMI goal changes with age. In people older than 70 years, BMI goal is 30-32 kg / m2 (**20**), data in correlation with another study carried out in Spain with 1,008 people over 65 years of age followed for 11.6 years, where it was observed that the ideal BMI was in the range of 30-35 kg / m2. Median survival in grade I obese patients was 13.8 years, in overweight subjects 12.3 years, in normal weight subjects 10 years, and in lean subjects 5.6 years. In this age group, obese people survived an average of nearly four years longer than normal-weight subjects (**21**). Another reason that could explain the MHO phenotype in the elderly population is physical condition. It is known that 30% of the obese population is healthy due to their physical condition. The physical condition of the patient is rarely taken into account based on his aerobic or cardiorespiratory capacities. Because of this, MHO subjects have a 38% lower risk of death from any associated cause, compared to obese subjects with metabolic disturbances. On the other hand, the risk of developing or suffering from cardiovascular disease or cancer associated with their weight is reduced by between 30% -50% in this population (**22**, **23**).

Few studies have analyzed the MHO phenotype in the elderly, as well as the impact of lifestyle modification (diet, physical activity) on OMICS (epigenetics and metabolomics) and on the components of the intestinal microbiota of these patients. On the other hand, the exposure through the diet to perfluorinated pollutants, bisphenol A, parabens and benzophenones has not been enough studied in the population and it is very interesting to know their role as endocrine disruptors. Due to the possible implication of different genes and proteins involved in the molecular mechanisms that regulate the growth, expansion and homeostasis processes of adipose tissue, new studies are being generated to investigate the relationship between obesity and insulin resistance, without a known pathogenic concept established (**24**).

In conclusion, the study of elderly subjects with MHO phenotype can help to test the hypothesis of the transitional nature of this condition. Studies on the underlying mechanisms that lead the population to reach old age without developing insulin resistance and the pro-inflammatory changes that characterize obesity are of great pathophysiological interest and may help to identify possible therapeutic targets. With this background, we set out the objective of the -omic study of different candidate genes, as well as the involvement of the intestinal microbiota and the analysis of certain endocrine disruptors, and their relationship with lifestyle in elderly patients with a MHO phenotype. The identification of these elderly people who present differences in their profiles could help us to better understand why some obese subjects maintain a state of insulin sensitivity. In fact, research in these fields can elucidate new strategies for the prevention and therapy of the development of insulin resistance and atherogenesis in the obese population.

References:

1. Comuzzie AG et al. J Mol Med 2001;79(1):57-70

2. Primeau V et al. Int J Obes (Lond). 2011;35:971-81.

3. Calori G et al. Diabetes Care. 2011;34:210-5.

4. Pajunen P et al. BMC Public Health. 2011;1;11:754.

5. Khawaja KI et al. Singapore Med J. 2018 Feb 12. doi: 10.11622/smedj.2018019.

6. Karelis AD et al. Diabetologia. 2008;51:1752-4.

7. Dalzill C et al. Can J Cardiol. 2014;30(4):434-40

8. Janiszewski PM & Ross R.. Diabetes Care. 2010;33:1957-9.

9. Perseghin G. Diabetologia. 2008;51:1567-9.

10. Karelis AD. Lancet. 2008;372:1281-3.

11. Tomiyama AJ et al. Int J Obes (Lond). 2016; 40(5):883-6

12. Lin H et al. Medicine (Baltimore). 2017;96(47):e8838.

13. Gomez-Huelgas R et al. Int J Obes (Lond). 2010;35:292-9.

14. Bernal-Lopez MR et al. Diabetes Res Clin Pract. 2011;93:e57-60.

15. Gomez-Huelgas R et al. Endocr Pract. 2013; 19 (5): 758-68

16. Goday A et al. BMC Public Health. 2016;16(1):248-62

17. Meigs JB et al. J Clin Endocrinol Metab. 2006;91:2906-12.

18. Childers D et al. Int J Obes 2010:34;1231-8

19. Willett WC et al. N Engl J Med. 1999;341(6):427-34.

20. Allison DB et al. Int J Obes 1997;21:424-31.

21. Zunzunegui M et al. J Aging Health 2012;24:29-47.

22. Ortega FB et al. Prog Cardiovasc Dis. 2015;58(1):76-86

23. Gregorio-Arenas E, et al. Maturitas. 2016;92:162-7.

24. Dumas ME et al. Proc Natl Acad Sci U S A. 2006;103(33):12511-6

**Study hypothesis**

H1. We postulate that the maintenance and/or weight loss produced by a change in lifestyle, mediated by changes in dietary habits and physical activity, and undertaken at the community level in the elderly Metabolically Healthy Obesity (MHO), will improve insulin sensitivity, compared to the non-responder group. Environment factors such as diet are able to modify the profile of epigenetic changes and, consequently, influence the development of obesityassociated diseases such as insulin resistance.

H2. We postulate that epigenetic modifications (on histones and in the promoter region) in genes involved in insulin resistance in obesity (epiobesigenes) are responsible for the development of the same, through the regulation of gene expression of genes regulating expansion ability, adipose tissue differentiation, adipogenesis, lipogenesis and inflammation.

H3. We postulate that the components of the microbiota have an immune function, a trophic function and digestive function. For this, interindividual variability exists depending on the composition of the flora, which can modify the energy value of food.

**Objectives:**

1. To analyze the demographic, anthropometric and lifestyle characteristics of the elderly MHO population.

2. To study the body composition and its relationship with the components of the diet in the elderly MHO population after a lifestyle intervention based on Mediterranean diet and physical activity recommendations.

3. To analyze the methylome and epigenetics involved in the pathways responsible for insulin resistance, inflammatory markers, lipid metabolism and adipokines. as well as the gene expression of methylases and how they affect the methylation profiles of an elderly MHO population.

4. To identify and study the alterations of methylome metabolites and the lipidomic pattern in an elderly MHO population undergoing a lifestyle modification intervention.

5. To explore the composition of the intestinal microbiota and its relationship with the components of the diet in the elderly MHO population.

6. To analyze the different endocrine disruptors and their relationship with lifestyle modification in the elderly MHO population.

**Study desing**

1. Open cross-sectional study that includes a MHOe population aged > 65 years of both sexes.
2. Inclusion criteria:
   1. Aged >65 years
   2. BMI ≥30-<40 kg/m2
   3. One or none of the following 4 cardio-metabolic disorders:
      1. Systolic blood pressure ≥140 mmHg and/or diastolic blood pressure ≥ 90 mmHg
      2. Triglycerides ≥150 mg/dL
      3. HDL-C <40mg/dL in men and <50mg/dL women
      4. Fasting blood glucose ≥100mg/dL, following the World Health Organisation criterion of MHO
3. Exclusion criteria:
   1. Diabetes
   2. Hypertension
   3. Previous cardiovascular disease (coronary, cerebrovascular or peripheral; aortic aneurysm, heart failure)
   4. Severe associated disease (advanced organ failure, dementia, cancer)
   5. Immobilized or terminally ill individuals
   6. Alcoholism or drug addiction
   7. Severe psychiatric illness
   8. Weight loss ≥5 kg in the last 6 months of unknown cause.
4. Recruitment and enrollment: recruitment will be carried out via visits performed to centers for the healthy elderly belonging to the Sports Area (Sport Medicine) of Malaga City Hall (Andalusia, Spain). Once the possible participants are selected, they are contacted and informed about the study design and objectives and summoned to the Department of Internal Medicine in the Regional University Hospital of Malaga. All participants in the study will give their written informed consent and protocols have been approved by the institutional ethics committee (Comité de Ética de la Investigación Provincial de Malaga (ref: PI18/00766-260718), belonging to the Andalusian Health Service).
5. Sample size: Simple Interactive Statistical Analysis (SISA) was used to calculate the sample size. We assumed a 95% confidence level (0.5% error), a statistical power of 80%, and a loss rate of 5%. The sample size needed is 150 patients.
6. Intervention: selected participants will follow a Mediterranean Diet advised by a nutritionist. The recommended caloric intake will be 1500-1750 kcal/day, distributed as follows: 30% of fats (5-8% of saturated fatty acids, 15-18% of monounsaturated fatty acids, 5-8% of polyunsaturated fatty acids and <300 mg of cholesterol/day), 55% of carbohydrates (< 10% of simple sugars, 40% complex sugars and low glycemic index) and 15% of proteins. The recommended Mediterranean Diet is based on the Trichopoulou criteria, prioritizing olive oil as the main cooking fat, consuming poultry or rabbit meat preferably rather than red meat and encouraging the consumption of fish, fruits, legumes and vegetables. Similarly, subjects will be encouraged to practice daily physical activity adapted to their age and physical condition, following the internationally-accepted physical activity guidelines (Physical Activity Guidelines for Americans. Chapter 5. Avalaible on: <https://health.gov/our-work/physical-activity/current-guidelines>). The intervention will last 24 months. Visits will be made at baseline and then at 4 and 12 months to reinforce the intervention and monitor the study variables and at 24 months to monitor for the last time the study variables.
7. Follow-up: medical visits will be performed at 4, 12 and 24 months after the start of the intervention. Anthropometric measurements will be taken, blood samples will be analyzed, and questionnaires will be completed in order to analyze the impact of the lifestyle intervention.
8. Outcomes:
   1. Primary outcome: composite outcome where the researchers will analyze the effectiveness of a multidisciplinary and multicomponent intervention carried out in the community setting, aimed at modifying the lifestyle (use of a Mediterranean diet and promotion of physical activity) to prevent the incidence of cardiometabolic alterations in elderly healthy metabolically obese subjects. This has been quantified as an improvement in Mediterranean diet adherence through food frequency questionnaires (baseline and 4, 12 and 24 months) and improvement in physical activity intensities, measured by accelerometry (minutes/day).

1. Nutritional assessment and monitoring: food intake analyzed using questionnaires at baseline and at 4, 12 and 24 months:

1.1. A non-consecutive, 3-day dietary record (two workdays and one weekend day), containing detailed information about food composition and cooking recipes over 72 hours

1.2. A food frequency questionnaire (number of times/day, number of days/week, number of days/14 days, number of days/month, rarely, or never)

1.3. Adherence to Mediterranean diet assessed by a validated 14-item food consumption frequency questionnaire

2. Physical activity monitored using a GENEActiv Actigraph GT3X+ accelerometer at baseline and at 4, 12 and 24 months. The accelerometer should be worn under the chest with a tight elastic belt to ensure close contact with the body. Recordings are made every day for at least 7 days (weekdays and the weekend) to take their hours of physical activity and sleep, except during water activities. Physical activity is also evaluated using the Rapid Assessment of Physical Activity (RAPA) questionnaire, a validated 7-item questionnaire at baseline and at 4, 12 and 24 months

**b.** Secondary outcome measures**:**

1. Anthropometric variables measured by trained personnel at baseline and at 4, 12 and 24 months:

1.1. Weight measured using an electronic scale: TANITA Body Composition Analyzer. Type TBF-300 MA. (TANITA Corporation; 1-14-2 Maeno-cho, Itabashi-ku. Tokio, Japan)

1.2. Height measured with no shoes using a wall stadiometer (Stadiometer Barys Electra Model. 511-300-A0A. ASIMED)

1.3. BMI calculated by dividing weight (kg) by height squared (m²)

1.4. The waist/hip index (WHI) calculated as the ratio of abdominal circumference (at the level of the mid-point between the anterosuperior iliac crest and the last costal arch, parallel to the ground and upon exhalation) and hip, both in cm

1.5. Blood pressure measured with a validated automated electronic sphygmomanometer (OMRON M7 (HEM-780-E, OMRON Healthcare Co. Ltd, Kyoto, Japan) after 5 minutes of rest while the participant is in a seated position

2. Serum adipokine and inflammatory biomarkers levels (IL-6 and TNFa) measured using an enzyme-linked immunosorbent assay (ELISA) (R&D Systems, Inc., Minneapolis, MN, USA) on blood samples collected after an overnight fast at baseline and at 4, 12 and 24 months

3. High-sensitivity CRP levels measured using ELISA (DRG Instruments GmbH, Germany) on blood samples collected after an overnight fast at baseline and at 4, 12 and 24 months
