# Supplementary material for: Metabolically healthy obesity: Inflammatory biomarkers and adipokines in elderly population
Source: PLoS One. 2022 Jun 9;17(6):e0265362. doi: 10.1371/journal.pone.0265362 (PMC9182320; doi:10.1371/journal.pone.0265362)
Supplement: Attachment — Submitted filename: Response to Reviewer 2.docx [file pone.0265362.s002.docx]

PONE-D-21-33113

Metabolically Healthy Obesity: Inflammatory biomarkers and adipokines in elderly population

PLOS ONE

**Reviewers' comments:**

Reviewer's Responses to Questions

**Comments to the Author**

**1. Is the manuscript technically sound, and do the data support the conclusions?**

The manuscript must describe a technically sound piece of scientific research with data that supports the conclusions. Experiments must have been conducted rigorously, with appropriate controls, replication, and sample sizes. The conclusions must be drawn appropriately based on the data presented.

**Reviewer #2:** Partly

**2. Has the statistical analysis been performed appropriately and rigorously?**

**Reviewer #2:** No

**3. Have the authors made all data underlying the findings in their manuscript fully available?**

The [PLOS Data policy](http://www.plosone.org/static/policies.action#sharing) requires authors to make all data underlying the findings described in their manuscript fully available without restriction, with rare exception (please refer to the Data Availability Statement in the manuscript PDF file). The data should be provided as part of the manuscript or its supporting information, or deposited to a public repository. For example, in addition to summary statistics, the data points behind means, medians and variance measures should be available. If there are restrictions on publicly sharing data—e.g. participant privacy or use of data from a third party—those must be specified.

**Reviewer #2:** Yes

**4. Is the manuscript presented in an intelligible fashion and written in standard English?**

PLOS ONE does not copyedit accepted manuscripts, so the language in submitted articles must be clear, correct, and unambiguous. Any typographical or grammatical errors should be corrected at revision, so please note any specific errors here.

**Reviewer #2:** Yes

**5. Review Comments to the Author**

Please use the space provided to explain your answers to the questions above. You may also include additional comments for the author, including concerns about dual publication, research ethics, or publication ethics. (Please upload your review as an attachment if it exceeds 20,000 characters)

**Reviewer #2:** The authors of this study explored the phenotype of metabolically healthy obesity. Multiple biomarkers of obesity and inflammation were assessed in response to lifestyle modifications. In my opinion, the authors do not use appropriate statistical methods in the study (e.g, Student t-test, assessments of normal distributions). A revision must include justification of statistical tests and apply post-hoc testing.

**Response: The aim of the study was to compare inflammation biomarkers at baseline and after 12 months of intervention. As it is explained in “Statistical Analysis” section, the authors noted that “**Quantitative variables with normal distribution were expressed as mean ± standard deviation (SD) and qualitative variables were expressed as percentages. Student t-test to compare quantitative variables and the Chi-square test to compare qualitative variables were used. Bivariate correlations were determined using Pearson correlation coefficient**.”**

**Authors consider that post-hoc testing is not applicable to this study as there are only 2 times of evaluation, as we compare baseline conditions Vs. 12 months after intervention (post-hoc tests need at least 3 groups).**

**6. PLOS authors have the option to publish the peer review history of their article (**[**what does this mean?**](https://journals.plos.org/plosone/s/editorial-and-peer-review-process#loc-peer-review-history)**). If published, this will include your full peer review and any attached files.**


If you choose “no”, your identity will remain anonymous but your review may still be made public.


**Do you want your identity to be public for this peer review?** For information about this choice, including consent withdrawal, please see our [Privacy Policy](https://www.plos.org/privacy-policy).

**Reviewer #2:** No

[NOTE: If reviewer comments were submitted as an attachment file, they will be attached to this email and accessible via the submission site. Please log into your account, locate the manuscript record, and check for the action link "View Attachments". If this link does not appear, there are no attachment files.]

While revising your submission, please upload your figure files to the Preflight Analysis and Conversion Engine (PACE) digital diagnostic tool, <https://pacev2.apexcovantage.com/>. PACE helps ensure that figures meet PLOS requirements. To use PACE, you must first register as a user. Registration is free. Then, login and navigate to the UPLOAD tab, where you will find detailed instructions on how to use the tool. If you encounter any issues or have any questions when using PACE, please email PLOS at [figures@plos.org](mailto:figures@plos.org). Please note that Supporting Information files do not need this step.
